# DNA repair mechanisms in cancer development and therapy

**DOI:** 10.3389/fgene.2015.00157

**Published:** 2015-04-23

**Authors:** Alessandro Torgovnick, Björn Schumacher

**Affiliations:** ^1^Institute for Genome Stability in Ageing and Disease, Medical Faculty, University of Cologne, Cologne, Germany; ^2^Cologne Excellence Cluster for Cellular Stress Responses in Aging-Associated Diseases Research Center, University of Cologne, Cologne, Germany; ^3^Center for Molecular Medicine Cologne, University of Cologne, Cologne, Germany; ^4^Systems Biology of Ageing Cologne, University of Cologne, Cologne, Germany

**Keywords:** DNA repair, cancer therapy, aging, genome instability, progeroid syndromes, xeroderma pigmentosum, ataxia telangiectasia, Fanconi anemia

## Abstract

DNA damage has been long recognized as causal factor for cancer development. When erroneous DNA repair leads to mutations or chromosomal aberrations affecting oncogenes and tumor suppressor genes, cells undergo malignant transformation resulting in cancerous growth. Genetic defects can predispose to cancer: mutations in distinct DNA repair systems elevate the susceptibility to various cancer types. However, DNA damage not only comprises a root cause for cancer development but also continues to provide an important avenue for chemo- and radiotherapy. Since the beginning of cancer therapy, genotoxic agents that trigger DNA damage checkpoints have been applied to halt the growth and trigger the apoptotic demise of cancer cells. We provide an overview about the involvement of DNA repair systems in cancer prevention and the classes of genotoxins that are commonly used for the treatment of cancer. A better understanding of the roles and interactions of the highly complex DNA repair machineries will lead to important improvements in cancer therapy.

## Introduction

Living organisms have the crucial task to preserve their genome and faithfully transmit it across generations. Transmission of genetic information is constantly in a selective balance between the maintenance of genetic stability versus elimination of mutational change and loss of evolutionary potential. The DNA molecule is under the continuous attack of a multitude of endogenous and exogenous genotoxic insults and it has been estimated that every cell experiences up to 10^5^ spontaneous or induced DNA lesions per day (De Bont and van Larebeke, 2004).

Endogenous damage can result from DNA base lesions like hydrolysis (deamination, depurination, and depyrimidination) and alkylation (6-*O*-Methylguanine) or oxidation (8-oxoG) by intracellular free radical oxygen species (ROS) that can occur as by-products of mitochondrial respiration (Lindahl and Barnes, 2000). Mutations can also arise during normal cellular metabolism for instance by erroneous incorporation of deoxyribonucleotides (dNTPs) during replication.

Environmental sources of damage can be physical [e.g., ultraviolet (UV) light, ionizing radiations (IRs), and thermal disruption] or chemical (e.g., chemotherapeutic drugs, industrial chemicals, and cigarette smoke) and their effects varies from the formation of cyclobutane pyrimidine dimers (CPDs) and pyrimidine 6-4 pyrimidone photoproducts (6-4PPs) following UV exposure, to the introduction of single and double DNA strand breaks upon IR treatment, or to inter- and intrastrand DNA crosslinks, which result from various chemotherapeutic drugs (Table 1; Ciccia and Elledge, 2010).

**TABLE 1:** **Distinct DNA repair systems are specialized to repair the various types of DNA lesions**.

Repair mechanism	Lesion feature	Genotoxic source (examples)
Base excision repair (BER)	Oxidative lesions	Reactive oxygen species (ROS)
Nucleotide excision repair (NER)	Helix-distorting lesions	UV radiation
Translesion synthesis	Various lesions	Various sources
Mismatch repair (MMR)	Replication errors	Replication
Single stand break repair (SSBR)	Single strand breaks	Ionizing radiation, ROS
Homologous recombination (HR)	Double-strand breaks	Ionizing radiation, ROS
Non-homologous end joining (NHEJ)	Double-strand breaks	Ionizing radiation, ROS
DNA interstrand crosslink repair pathway	Interstrand crosslinks	Chemotherapy

**FIGURE 1:**
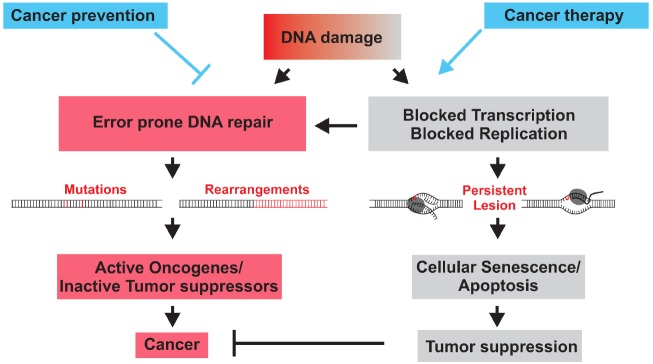
**DNA damage causes cancer development when erroneous DNA repair leads to mutations of chromosomal aberration that activate oncogenes or inactivate tumor suppressors genes (red).** When DNA damage persists and interferes with replication or transcription, DNA damage checkpoints trigger cellular senescence or apoptosis that inactivate or eliminate damaged cells and thus suppress tumorigenesis (gray). DNA repair mechanisms prevent cancer by preventing mutations. Chemo- and radiotherapy often inflict DNA damage to halt cancer cell proliferation or trigger the apoptotic demise of cancer cells.

DNA lesions can alter the primary structure of the double helix thereby affecting transcription and replication. Erroneous repair of lesions can lead to mutations in the genome that can be inherited to daughter cells with deleterious consequences for individual’s health. As a consequence, eukaryotic cells have evolved a complex signaling network of repair processes known as the DNA damage response (DDR). The importance of DNA repair mechanisms is highlighted by the existence of many devastating human syndromes that are caused by defects in DDR genes. Notably, many of these mutations generally display increased sensitivity to DNA damaging agents and predispose to the development of specific cancer types (Curtin, 2012). Already Theodor Boveri recognized cancer as a disease of the genome. Indeed mutations and chromosomal aberrations can lead to alterations in the gene function. Uncontrolled tumorous cell growth occurs when oncogenes are activated or tumor suppressor genes inactivated (Figure 1). The underlying role of DNA damage in cancer development has become particularly evident when genetic defects in DNA repair systems lead to increased cancer susceptibility.

## DNA Repair Defects Lead to Tumor Development

### Xeroderma Pigmentosum

Xeroderma pigmentosum (XP), Cockayne syndrome (CS), and trichothiodystrophy (TTD) are rare autosomal recessive diseases caused by defects in the nucleotide excision repair (NER) pathway that protects the DNA molecule from the damage inflicted by UV irradiation (Cleaver, 2005). Indeed, XP was initially described by the dermatologists Hebra and Kaposi (1874) and was the first syndrome associated with a defect in a DNA processing pathway (Cleaver, 1968).

The NER-associated diseases share an increased sun-sensitivity and freckling in the skin areas exposed to the sun but while XP is a skin cancer-prone (>1000-fold increase) disease (basal cell cancer, squamous cell cancer, and malignant melanoma; Kraemer et al., 1987), CS and TTD are not.

Bypass of unrepaired DNA lesions during replication in dividing cells of XP patients can lead to mutations. Mutations can alter the sequence and consequently the function of tumor suppressors and oncogenes. Consequently, XP patients bear not only a highly elevated risk for developing skin cancer but also a >10- to 20-fold increase of internal malignancies like leukemias, brain and lungs tumors before the age of 20 (Bootsma et al., 2001).

XP patients present differences in sunburn reaction that inversely correlate with cancer risk: 60% of the cases have an extreme UV light sensitivity directly after birth while the remaining 40% only show visible signs from the age of 2 years where a freckle-like pigmentation becomes more evident on the face. Paradoxically, the latter ones have higher risk to develop cancer. XP patients can also present, in about 20–30% of cases, neurological abnormalities (Diderich et al., 2011).

Complementation studies from fibroblasts derived from XP patients have shed light on the fundamental players involved in this pathway: mutations in seven different NER genes [from Xeroderma pigmentosum, complementation group A (XPA) to Xeroderma pigmentosum, complementation group G (XPG); De Weerd-Kastelein et al., 1972] plus a variant form, Xeroderma pigmentosum, complementation group V (XPV), defective in the translesion DNA polymerase eta (Lehmann et al., 1975), lead to XP.

Nucleotide excision repair repairs the major lesions caused by UV light, the CPDs and 6-4PPs that distort the DNA double helix. A similar type of damage, as well resolved by NER, is caused by polycyclic aromatic hydrocarbons (tobacco smoke or DNA crosslinking agents like Cisplatin or Benzopyrene; Leibeling et al., 2006) and ROS-generated cyclopurines. NER comprises two subpathways: global genome-NER (GG-NER) that scans the entire genome for helix-distorting lesions and transcription coupled-NER (TC-NER), which is operating only on actively expressed genes and is activated when RNA polymerase II stalls at a lesion. The NER mechanism consists of four main different steps: damage recognition, DNA unwinding around the lesion, cleavage and excision of the damaged strand and synthesis of the new DNA with concomitant final ligation. The only difference between the two NER branches resides in the DNA damage recognition phase and for the fact that TC-NER is faster than GG-NER in damage resolving (Hanawalt and Spivak, 2008).

In the first step of GG-NER, the protein complexes XPC-HHR23B-Centrin2 and XPE-DDB2 sense the damage and initiate the repair process by recruiting other NER factors. The multiprotein complex transcription factor IIH (TFIIH; TFIIH subunits: XPB, GTF2H1 GTF2H2, GTF2H3, GTF2H4, XPD, MNAT1, CDK7, CCNH, GTF2H5) generates a transiently open DNA structure by using the 3′-5′ and 5′-3′ nuclease activity of the two ATP-dependent helicases XPB and XPD (Evans, 1997). The fundamental role of these proteins is underline by the fact that XPB and XPD knockout mice are not viable (Cleaver, 2005). XPD is required not only for its helicase unwinding capacity but also to verify the damage after XPC loading. The Arch and Fe-S cluster domains of XPD form a channel where the damaged DNA is scanned in a 5′-3′ direction. After unwinding of a 27–30 bp DNA tract, the exposed filament is completely covered by the replication protein A (RPA; de Laat et al., 1998). RPA, together with XPA loading on the 5′ side of the lesion (Krasikova et al., 2010), is involved in the correct positioning of the endonucleolytic cleavage mediators. The incision step is carried out by two structure specific endonucleases respectively named XPF-ERCC1 and XPG. The first cut at the 5′-end of the lesion by XPF-ERCC1 is than followed by the action of XPG on the opposite DNA filament (Fagbemi et al., 2011). By using the complementary strand as a template, DNA polymerase *δ* (in non-replicating cells) and *ε* (in dividing cells) synthesize the new error-free sequence starting from the 3′-hydroxyl extremity generated by XPF-ERCC1. The remaining 3′-end incision is finally closed by Ligase I or III (Moser et al., 2007).

The TC-NER subpathway initiates when RNA polymerase stalls at a DNA lesion. XPC, which without XPE is incapable of binding to CPDs (Fitch et al., 2003; Sugasawa et al., 2005), is dispensable for TC-NER (Venema et al., 1991). Upon RNAPII stalling the Cockayne syndrome protein B (CSB) recruits Cockayne syndrome protein A (CSA), whereupon the same NER core machinery is activated as following GG-NER-mediated damage recognition. In 80% of the cases CS patients have mutations in CSB (Natale, 2011) and show neurodegeneration and cachectic dwarfism. A possible explanation for the lack of tumors observed in CS patients is the high susceptibility of CS-derived cells to undergo cell death after DNA damage (McKay et al., 2001). In addition, it was shown that CS mouse models exhibit reduced levels of circulating growth factors such as IGF-1 (van der Pluijm et al., 2007), suggesting that a reduced endocrine growth environment might prevent cancer development (Schumacher et al., 2008).

### Ataxia Telangiectasia

The major regulators of the DDR are the two serine-threonine kinases ATM [ataxia telangiectasia (AT) mutated] and ATR (ATM and RAD3-related) which both belong, together with SMG-1 (suppressor of mutagenesis in genitalia), DNA-PKcs (DNA-dependent protein kinase catalytic subunit) and mTOR (mammalian target of rapamycin), to the phosphonositide 3-kinase (PI3K)-related protein kinases (PIKKs) family. All of them share a conserved C-terminal kinase domain structure flanked by the FAT and FATC domains, two conserved regions, with high sequence similarity, regulating the kinase activity (Cimprich and Cortez, 2008).

The overlapping substrates of ATM and ATR comprise more than 700 different proteins mainly involved in DNA repair, cell cycle arrest, and transcription but also in developmental processes, immunity and intracellular protein traffic (Matsuoka et al., 2007). Among the most important, ATM and ATR respectively target the two serine-threonine protein kinases: checkpoint kinase 2 (CHK2) and CHK1, that function as key signal transducers of the DDR (Bartek and Lukas, 2003). In contrast to ATM and CHK2, the ATR and CHK1 kinases are indispensable for the viability of mammalian cells (Brown and Baltimore, 2000; de Klein et al., 2000).

Humans carrying homozygous mutations (0.5–1%) in the ATM gene (432 mutations have been reported without any hotspots and generally lead to protein instability—Leiden Open Variation database) suffer from the neurodegenerative disease AT, which is characterized by radiation sensitivity, chromosomal instability and predisposition to cancer. Up to 30% of AT patients develop lymphoid tumors since ATM play a critical role in the differentiation of T and B cells (Lumsden, 2004). Carriers of heterozygous missense mutations leading to the expression of inactive but stable variants acting as dominant ATM version against the wild type allele have higher incidence to develop breast, colorectal and stomach cancer (Thompson et al., 2005; Paglia et al., 2009).

Hypomorphic mutations in ATR lead to Seckel syndrome. The main features of this disease are growth retardation, microcephaly and a characteristic “bird-headed” facial appearance (O’Driscoll et al., 2003). While germline ATR mutations have not yet been reported, ATR was recently found to be downregulated in head and neck cancers (Moeller et al., 2011) and mutations within the FAT domain were observed in oropharyngeal-tumor tissue (Tanaka et al., 2012).

Although they share many substrates, ATM and ATR are activated in different ways. ATR is mainly induced upon DNA single strand breaks (SSBs) originated by replication fork stalling or as result of double strand breaks (DSBs) processing and NER activity. On the other hand, ATM primarily responds to DSBs caused by IR or ROS as well as breaks coming from physiological processes like meiosis, telomere maintenance, or immune system maturation (assembly of the T cell receptor and immunoglobulin genes via V(D)J recombination; Shiloh, 2003).

In the ATR activation process: RPA, after coating the single strand DNA, recruits the ATR interacting protein (ATRIP). This complex helps to localize the site of damage (Zou and Elledge, 2003) and to direct the loading of the RAD9-RAD1-HUS1 (9-1-1) clamp through the interaction with the RAD17-replication factor C (RFC). After 9-1-1 is loaded on the 5′ end of the ssDNA, the ATR activator topoisomerase-binding protein-1 (TOPBP1) can be recruited and activates ATR in an ATRIP-dependent manner (Cimprich and Cortez, 2008). Another mediator of ATR activation is Claspin (Smits et al., 2010). Activated ATR phosphorylates CHK1 on Ser317 and Ser345 residues. Additional substrates of ATR phosphorylation include: ATRIP, Rad17, Rad9, TopBP1, Claspin, H2AX, WRN, BLM, BRCA1 (breast cancer susceptibility gene 1), and FANCD2 (Cimprich and Cortez, 2008).

Ataxia telangiectasia mutated is found in the nucleus of undamaged cells in the form of inactive dimers or higher order multimers, configuration that inhibit, by masking with the FAT domain, the kinase domain. Upon DNA damage, ATM undergoes autophosphorylation on residues Ser367, Ser1893, and Ser1981 with the last one located within the FAT domain. These posttranslational modifications result in dimer dissociation and release of active kinase monomers (Bakkenist and Kastan, 2003; Kozlov et al., 2006). Upon formation of a DSB, the sensor complex MRE11-RAD50-NBS1 (MRN), which is composed by the meiotic recombination protein 11 (MRE11), the DNA repair protein RAD50 and the Nijmegen breakage syndrome protein-1 (NBS1), localize to the damaged area together with ATM (Lee, 2004). Recently it was reported that ubiquitination of NBS1 by SCF-Skp2 E3 ligase trigger the recruitment and activation of ATM on DSB formed upon IR treatment (Wu et al., 2012). Activated ATM phosphorylates the histone variant H2AX, which is then bound by the mediator of DNA damage checkpoint protein-1 (MDC1). MDC1 induces the recruitment of other ATM–MRN complexes resulting in the establishment of a positive feedback-loop that leads to further H2AX phosphorylation and amplification of the initial signal (Lavin et al., 2005). The pool of activated ATM within the cell appear to be divided in two fractions: the first one is physically bounded to DSB sites while the other one is free to reach other targets that required to be activated (Shiloh, 2003).

Ataxia telangiectasia mutated exerts its survival function through the induction of cell cycle checkpoints. In the G1-S checkpoint, ATM phosphorylates the tumor suppressor p53 on S15 leading to the disruption of the inhibitory association with MDM2. Activated p53 induces p21, which binds to and inhibits the S-phase-promoting Cdk2-CyclinE complex (Sancar et al., 2004).

During the G2-M checkpoint, ATM phosphorylates monomers of CHK2 on Thr68 allowing the formation of CHK2 dimers that have as a main target the cell division cycle 25 homolog A (CDC25A). Phosphorylated CDC25A can finally be degraded by the proteasome and prevents cyclin-dependent kinase 2 (CDK2) and CDK1 dephosphorylation, which is required for progression through the cell cycle (Cimprich and Cortez, 2008).

### Fanconi Anemia

DNA inter- and intrastrand crosslinks represent a dangerous form of damage blocking vital cellular processes like transcription and replication. The Fanconi anemia (FA) pathway is responsible to repair these aberrations arising in the DNA structure as a result of chemotherapeutics drugs treatment, like cisplatin or mitomycin C (van der Heijden et al., 2004), or naturally evolved due to the interaction with lipid peroxidation products such as malondialdehyde (Stone et al., 2008). FA is an autosomal recessive disease that affects 1 every 100,000 births (Rosenberg et al., 2011) and it is characterized by growth retardation, infertility, bone marrow failure and susceptibility to acute myeloid leukemia. Solid tumors like head and neck, kidney, liver, medulloblastoma, gynecological, oesophageal, and skin cancers are also common between FA patients (Cerbinskaite et al., 2012).

Fanconi Anemia is a heterogeneous genetic disease, 16 different genes are involved in the establishment of the disorder and they can be divided in three major groups: the FA core complex, the I-D2 complex and downstream FA proteins. Eight proteins form the core complex, respectively named FANCA, FANCB, FANCC, FANCE, FANCF, FANCG, FANCL, and FANCM while the I-D2 complex is constituted by FANCD2 and FANCI (Walden and Deans, 2014). In the initial phase of the process, FANCM, which forms and heterodimer with FAAP24 (FA-associated protein 24 kDa), recognizes DNA interstrand cross-links (ICL) lesions and recruits other FA factors to the damaged site, the stalled replication fork. The association of FANCM with the chromatin is strengthened by histone fold protein 1 (MHF1) and 2 (MHF2; Singh et al., 2010) and it is followed by ATR activation (Schwab et al., 2010). Monoubiquitination of FANCD2 on Lys 561 and FANCI on Lys 523 by the core complex, which essentially constitutes a multisubunit E3 ubiquitin ligase, is the key step in the activation of the FA pathway (Taniguchi et al., 2002; Smogorzewska et al., 2007).

Despite the fact that FANCD2 was shown to have intrinsic nuclease activity (Pace et al., 2010), other nucleases are involved in the FA pathway and which one is responsible to perform the first cut and start unhooking the crosslinked DNA is still unknown. The best candidate to assume this function seems to be SLX4 (FANCP), which is a multidomain scaffold protein directed toward branched DNA and Holliday junction (HJ) structures and able to interact with three distinct nucleases: SLX1, XPF-ERCC1, and MUS81-EME1. The interaction between SLX4 is with the NER endonuclease XPF-ERCC1 was indeed shown to be crucial for the removal of ICLs (Crossan et al., 2011). FAN1 (FA-associated nuclease 1) is another nuclease recruited to the damaged site by ubiquitinated FANCD2. FAN1 abrogation does not affect ICLs-induced DSBs formation most likely resembling the possibility that FAN1 is required further down in the steps of the repair process (Kratz et al., 2010).

The FA pathway allows resolving the replication fork stalling by inducing the formation of a DSB and by coordinating the action of three critical repair mechanisms: translesion synthesis (TLS) bypasses the lesion and, after toxic adducts removal by NER, the gap is closed by homologous recombination (HR). The ID complex is finally able to leave the previously damaged area thanks to deubiquitination mediated by USP1 (ubiquitin specific peptidase 1) and UAF1 (USP1-associated factor 1; Nijman et al., 2005).

Although further work is required to fully understand each steps of the FA pathway, some of the downstream players involved are: FANCJ (BRIP1), DNA-dependent ATPase and 5′-3′ DNA helicase able to interact with BRCA1; FANCD1 (BRCA2), able to bind ssDNA and dsDNA and to stimulate RAD51 action; FANCN (partner and localizer of BRCA2, PALB2), required for FANCD1 stabilization and for the recruitment of BRCA2 and RAD51; and FANCO (RAD51C) involved in HJ resolution (Kottemann and Smogorzewska, 2013).

The tumorigenesis of FA is difficult to interpret due to the overlapping functions of all the aforementioned proteins working also in homology-directed repair. Of note, the FA pathway is also active in physiological conditions by preserving the replication fork stability during S-phase (the I-D2 complex was found to be ubiquitinated in undamaged cells; Schlacher et al., 2012) and acts as a barrier against error-prone repair processes such as non-homologous end joining (NHEJ). Accordingly, genomic instability, a typical feature of FA patients, was rescued in *C. elegans*, DT40 chicken and mammalian cells by inhibiting NHEJ components (Adamo et al., 2010; Pace et al., 2010).

### Breast Cancer Susceptibility Gene 1 and 2 (BRCA1 and 2)

Double strand breaks are the most threatening forms of DNA damage, if left unrepaired they can lead to chromosomal rearrangements or to cell death. To counteract DSBs, cells have evolved two different repair mechanisms: HR and NHEJ. HR is an error-free way to repair DSBs which takes place during S and G2 phases of the cell cycle where a sister chromatid is used as a homologous template (Roy et al., 2011). *Vice versa*, NHEJ, which fuses two broken chromosomal ends, can be mutagenic and can act independently of the cell cycle status (Caestecker and Van de Walle, 2013).

Two different ways of HR repair coexist: the classic model and the alternative synthesis-dependent strand-annealing (SDSA) model. In the first one, also known as Double HJ model, the 5′ and 3′ ends of a DSB are resected by nucleases (endonuclease Sae2, exonuclease Exo1, helicases Sgs1, and Dna2) and the 3′ ssDNA filament invades the intact sister chromatid, which is used as a template to repair the lesion. The displacement of the second strand results in the formation of a D-loop. The extension of the 3′ invading strand transforms the D-loop to a cross-shape structure known as HJ. The second 3′ overhang, not involved in the initial strand invasion, also produces a HJ with the homologous chromosome. This way of repair may result in the formation of chromosomal crossovers and principally takes place during meiosis (Helleday et al., 2007). To avoid the production of crossover in somatic cells, event that will end up in loss of heterozygosity (LOH), the double HJ can be dissolved by bloom helicase (BML) and Topoisomerase III (Wu and Hickson, 2003).

The SDSA process shares all the steps of the classic HR repair model except for the absence of the D-loop structure formation (Sung and Klein, 2006). SDSA always leads to non-crossover products and is supposed to be the most used way of HR in mitosis.

Both BRCA1 and BRCA2 are involved in the HR pathway. *BRCA1* and *BRCA2* mutations are found in approximately 5–7% of all hereditary breast cancers (Roy et al., 2011). In mice, homozygous BRCA1 and BRCA2 knockouts die at day 8–9 of development (Hakem et al., 1996; Suzuki et al., 1997).

Breast cancer susceptibility gene 1 plays major roles in different DNA repair mechanisms. It acts in HR, NHEJ and single-strand annealing (SSA) through its different interaction domains. Located at the N-terminus, the RING domain is the site for the interaction with BARD1 (BRCA-associated RING domain 1), a structurally-related protein responsible for BRCA1 stabilization and activity (Wu et al., 1996). The BRCA1/BARD1 heterodimer possesses E3 ubiquitin ligase activity and, upon DNA damage, mediates downstream signaling events through ubiquitination of other DDR targets including CtIP, H2AX, RNAPolII, and CstF (Caestecker and Van de Walle, 2013). At the C-terminus, BRCA1 has a domain shared between many DDR proteins: the BRCT1 domain (BRCA1 C-terminal), required for binding phosphorylated proteins during the DDR (Koonin et al., 1996) and essential for transcriptional regulation and chromatin unfolding (Monteiro, 2000; Ye et al., 2001). In the central part of the protein we find the DNA binding domain (DBD), the nuclear localization and exporting sequences and, most importantly, the serine-glutamine (SQ) and threonine-glutamine (TQ) motifs which are indispensable for BRCA1 activation through ATM/ATR phosphorylation (Caestecker and Van de Walle, 2013). Most of the cancer-associated BRCA1 mutations are found in the RING and BRCT domains (Roy et al., 2011).

Breast cancer susceptibility gene 1 is a component of three different multiprotein complexes involved in all cell cycle checkpoints: the BRCA1A complex (composed of Abraxas, BARD1, RAP80, BRCC36, BRCC45, and MERIT40), responsible to recruit BRCA1 to damaged sites; the BRCA1B complex (formed with BRIP1 and TOPBP1), mainly associated with replication-coupled DNA repair and the BRCA1C complex (formed together with CtIP and the MRN complex), which promotes HR despite NHEJ (Huen et al., 2010).

Interestingly, *BRCA1/BARD* mutations cannot only fuel genome instability due to impaired HR activity, but also promote genome stability as recently shown in *C. elegans* mutants of the *smc-5/6* complex that leads to replicative impediments and DSB formation at stalled replication forks (Wolters et al., 2014). The genome instability in *smc-5/6* mutants could be revered upon inactivation of the *BRCA1/BARD* complex. It is tempting to speculate that mutations in *BRCA1* might be sustained in the human genome as under certain conditions of replication fork breakdown prevention of HR could benefit genome stability.

The BRCA2 protein was recently purified and functionally validated by three independent research groups (Jensen et al., 2010; Liu et al., 2010; Thorslund et al., 2010). In contrast to the multiple functions of BRCA1, BRCA2 main role is to mediate the recruitment of RAD51 to DSBs during HR. BRCA2 carries, in the central part of the protein, a DBD able to bind both single and double stranded DNA and eight BRC repeats indispensable for the interaction with RAD51. Cancer-associated BRCA2 point mutations are found between these repeats (Venkitaraman, 2009).

Breast cancer susceptibility gene 2 prevents RAD51 binding to dsDNA and specifically direct it to ssDNA where it displace RPA (Thorslund et al., 2010). The PALB2, also known as FANCN, is the connection between BRCA1 and BRCA2. PALB2 is required for the colocalization of BRCA1, BRCA2, and RAD51 to the damaged sites and its dysfunction leads to severe HR defects (Zhang et al., 2009a).

### Mismatch Repair

The critical role of mismatch repair (MMR) in tumorigenesis is highlighted by the fact that loss of expression of MMR proteins predispose to colorectal, gastric, endometrial and ovarian cancers and inherited defects in the MMR genes are associated with the most prevalent cancer syndrome in humans, the Lynch syndrome (LS), previously known as hereditary nonpolyposis colorectal cancer (HNPCC; Guillotin and Martin, 2014). Moreover, MMR deficiency is present in 15% of all primary cancers (Furgason and Bahassi el, 2013).

The MMR pathway recognizes base–base mismatches and insertion-deletion loops (IDLs; Jiricny, 2006) originating from base misincorporation, tautomeric shifts, slippage of DNA polymerases, damage that acts as mismatch, and recombination duplex. The sequential events in MMR repair comprise damage recognition, excision, and resynthesis steps (Hsieh and Yamane, 2008). The MutSα and MutSβ complexes are the MMR lesion detectors. The first complex is composed by MSH2 and MSH6 and recognizes single base-base mismatches and 1–2 bp IDLs while the second one, formed by the MSH2 and MSH3 proteins, principally find and repair 2–12 bp IDLs (Iyama and Wilson, 2013).

Upon DNA binding, one of the three different heterodimeric complexes MutLα (MLH1-PMS2), MutLβ (MLH1-MLH3), and MutLγ (MLH1-PMS1) can be recruited to form, with MutS, a ternary structure. The complex formed with MutLα is the most important in the MMR pathway, is able to translocate in both directions along the damaged area and to recruit proliferating cell nuclear antigen (PCNA), RFC, and EXO1 to perform the excision step (Guillotin and Martin, 2014). MutLβ function is currently unknown whereas MutLγ is involved in meiotic recombination (Zhang et al., 2005). After damage resection, resynthesis is carried out by DNA polymerase *δ* and sealing of the nick by DNA ligase I (Larrea et al., 2010).

Being part of the replication fork, the MMR machinery operates mostly in dividing cells (Wagner and Meselson, 1976), nonetheless few publications report an active presence of MMR in the brain (Brooks et al., 1996).

Mismatch repair dysfunction accounts for the mutator phenotype in which base substitution and frameshift mutations are highly increased due to microsatellite instability (MSI). Microsatellites are short tandem repeated DNA sequences of 1–4 base nucleotides spread all over the genome. Replication of these repeats has high error risk and when they are present in tumor suppressor genes, a defective repair may have detrimental consequences (MSI; Guillotin and Martin, 2014).

## DNA Damaging Agents in Tumor Therapy

Cancer therapy was jumpstarted at the end of the Second World War by serendipity resulting from some of the darkest chapters of chemical warfare that brought so much suffering during the First World War. Already in the trenches of the First World War bone marrow suppression and lymphoid aplasia were reported upon exposure to the chemical warfare sulfur mustard. The critical link to its therapeutic potential became evident a few decades later when the secret load of the American vessel *S.S. John Harvey* was unleashed in the Italian harbor of Bari during a German air raid. Physicians detected reduced white blood counts in autopsies following the incidence. It turned out that the vessel’s load of nitrogen mustard had attacked the white blood cells suggesting that leukemias could be targeted by nitrogen mustard therapy. Already a few years later the first alkylating agents were introduced to cancer therapy. Strikingly, it was found that effective chemotaxis such as nitrogen mustard and cisplatin evoke damage in nuclear DNA that then results in cell death. Therefore, DNA damage not only causes tumor development but could also battle cancers by impairing cancer growth and ultimately triggering the death of malignant cells (Figure 1).

### Cisplatin

Also known as Peyrone’s chloride, cisplatin (*cis*-diamminedichloroplatinum) is one of the most widely used chemotherapeutic drugs. Its antitumor potential was discovered in the sixties by Rosenberg et al. (1965) when he accidentally found out that this metal salt was able to inhibit *Escherichia coli* cell division. Cisplatin soon drew interest in the scientific community and, after its efficacy was proven in mouse models (Rosenberg et al., 1969), it entered clinical trials and was finally approved by FDA in 1978 as a chemotherapeutic drug for the treatment of testicular and bladder cancers (Kelland, 2007b). The therapeutics properties of cisplatin were then extended to many other types of cancer including small and non-small cell lung, head and neck, ovarian, cervical, and colorectal (Lebwohl and Canetta, 1998; Galanski, 2006).

Once in the cytoplasm, cisplatin gets activated upon reaction with water, which can substitutes one or both the two *cis*-chloro groups of the molecule. The mono aquated form of cisplatin is the most reactive one, it can react with many cytoplasmic nucleophiles substrates including reduced glutathione (GSH), methionine and metallothioneins (MT) but its cytotoxic effect comes from the capacity to target DNA (Galluzzi et al., 2011). Inside the nucleus, cisplatin attacks the N7 nucleophilic site of purine bases leading to the formation of monofunctional adducts. Such adducts are able to form intra-strand crosslinking structures [90% 1,2 d(GpG) and 10% 1,2 d(ApG)] which represent the major type of DNA damage exerted by this chemotherapeutic drug (Dasari and Tchounwou, 2014). Cisplatin-mediated damage arrests cells in the G2 phase of the cell cycle and concomitantly triggers the activation of DNA repair pathways. If the damage is too severe, programmed cell death will be induced through the ATM/ATR/TP53 pathway (Damia et al., 2000; Pabla et al., 2008). Although cisplatin is a really potent apoptotic inducer, intrinsic or acquired resistance can represent an obstacle for its use in tumor therapy. Moreover, cisplatin resistance can either take place before or after DNA binding.

The copper transporter 1 (CTR1) regulates cisplatin cellular uptake. Cisplatin treatment of *Ctr1*^–/–^ mouse embryonic fibroblasts (MEFs) is associated with a reduced intracellular accumulation respect to wild type MEFs (Holzer et al., 2006) and CTR1 downregulation is found in cisplatin-resistant lung cancer cell lines (Song et al., 2004). Copper pretreatment of cochlear derived HEI-OC1 cells reduced cisplatin cytotoxicity (More et al., 2010). In addition to copper transporters, also organic cation transporters (OCTs) were recently discovered to be involved in cisplatin intake. Even if the uptake is the main cause of altered intracellular cisplatin level, the efflux process must be considered as well. The ABC ATPases-like multidrug resistance proteins (MRP) MRP1, MRP2, MRP3, MRP5, and the copper ATPases ATP7A and ATP7B mediate cisplatin export and were found to have altered expression in cisplatin resistant tumors (Burger et al., 2011).

Cisplatin resistance can also be established through the interaction with intracellular thiol-containing molecules such as GSH and MT. They can both sequester cisplatin within the cytoplasmic compartment and correlations between their expression level and cisplatin resistance were found in ovarian, cervical, lung, and bladder cancer cell lines (Köberle et al., 2010).

Cisplatin-induced DNA damage is primarily repaired by the FA pathway (Deans and West, 2011), as well as by NER and MMR. Enhanced activity of these repair mechanisms can promote cisplatin resistance. Indeed, higher and lower expression levels of the NER endonucleases ERCC1 and XPF were respectively found to be associated with resistance and sensitivity in ovarian and testis cancer cell lines (Köberle et al., 1999; Ferry et al., 2000; Welsh et al., 2004). Moreover, siRNA mediated downregulation of the ERCC1-XPF complex renders lung, ovarian and breast cancer cells more prone to death after cisplatin treatment (Arora et al., 2010).

Like NER, also MMR deficiency compromises cisplatin-induced apoptotic signaling (Topping et al., 2009) and it was observed to be always associated with an increased translesion synthesis (TLS) activity (Jung and Lippard, 2007). The specialized TLS polymerases are therefore another critical target to overcome resistance in patients carrying MMR mutations.

While cisplatin has a strong anti-cancer activity, it also exerts negative side effects like nephro- and neurotoxicity (Kelland, 2007a). The negative aspects and the concomitant possibility to acquire resistance after a certain period of treatment have pushed researchers, during the last 40 years, to design new platinum based drugs.

Approved by FDA in 1989, Carboplatin has, instead of the two *cis*-chloro groups, a bidentate dicarboxylate ligand, which slow down reactivity and unfavorable side effects. Carboplatin is actually used in the treatment of ovarian, head and neck, and lung tumors (Dasari and Tchounwou, 2014). The adducts formed by this molecule are the same ones introduced by cisplatin (Harrap, 1985) and thrombocytopenia is its main negative side effect.

The last platinum drug approved by FDA in 2002 is oxaliplatin. The large 1,2-diaminocyclohexane ligand plus the oxalate leaving group confers to oxaliplatin completely new characteristics: it is less dependent on the CTR1 transporter (Holzer et al., 2006) and forms DNA adducts which are not recognized by MMR (Fink et al., 1996). Apart from being effective in the treatment of cisplatin and carboplatin-resistant tumors (Raymond et al., 2002), oxaliplatin, in combination with 5-fluorouracil, is successfully employed in colorectal cancer treatment (FOLFOX therapy; Kelland, 2007b).

Between the recently developed platinum based drugs, phenanthriplatin is one of the most promising. This new compound kills cancer cells more efficiently than cisplatin and oxaliplatin and appear to be immune to acquired resistance mechanisms (Park et al., 2012).

### Nucleoside Analogs

Nucleoside analogs are anticancer metabolites that were developed based on modifications of physiological purine (adenosine, guanosine, inosine) and pyrimidine (cytidine, thymidine, uridine) nucleosides, the fundamental precursors of ATP, DNA, and RNA. This class of drugs is widely used in hematological malignancies and solid tumors and, as well, for the treatment of viral infections (Galmarini et al., 2002).

Nucleoside analogs exert their cytotoxic activities after being incorporated into DNA and RNA molecules leading respectively to replication and transcription inhibition, or by directly interfering with critical enzymes such as polymerases, kinases, ribonucleotide reductases, methyltransferases, nucleoside phosphorylases, and thymidylate synthases (Jordheim et al., 2013). The cellular uptake of these hydrophilic antimetabolites is mediated by two major families of nucleoside transporter (NT) proteins: the equilibrative NTs (ENTs) and the concentrative NTs (CNTs; Zhang et al., 2007). Within the cell, the same enzymes [deoxycytidine kinase (dCK), deoxyguanosine kinase (dGK), thymidine kinase 1 (TK1) and 2 (TK2)] that are responsible for providing dNTPs for DNA synthesis in resting cells sequentially phosphorylate nucleoside analogs to mono, di- and tri-phosphate variants. Triphosphates represent the active cytotoxic form of nucleoside analogs (Jordheim and Dumontet, 2007).

Targeting every proliferating cell, the lack of specificity of nucleoside analogs leads to negative side effects ranging from bone marrow suppression with immune system depletion to neurotoxicity. Concomitantly, targeted cells can also develop resistance to nucleoside analogs due to decreased activity of the dCK/dGK activating enzymes or by loss of expression of the NTs.

Cytarabine or ara-c was the first nucleoside analog developed starting from modification of 2-deoxycytidine and approved by FDA in 1969 for acute myeloid leukemia (AML) treatment (Johnson, 2001). Ara-c carries a hydroxyl group inserted at the 2′ position of the sugar and, once inside the cell, becomes phosphorylated by dCK. The triphosphate form, ara-CTP, can be inserted into the DNA in active synthesis instead of deoxycytidine triphosphate (dCTP). Since the 3′–5′ proofreading activity of DNA polymerases is slower than ara-CTP incorporation, the modified newly inserted nucleoside, which is not a good 3′ substrate for DNA polymerases, will lead to the stalling of the replication fork (Ross et al., 1990). Gemcitabine is also a 2-deoxycytidine analog with two fluorine introduced in the 2′ position of the sugar. Like cytarabine, the antitumor activity of this molecule is due to the incorporation of the triphosphate form into DNA and concomitant competition with dCTP (Hertel et al., 1990). Gemcitabine has the capacity to inhibit ribonucleotide reductase and therefore decreasing the deoxynucleotide pools (Wang et al., 2007). This nucleoside analog is active in solid tumors such as pancreatic, breast, ovarian and non-small cell lung cancers (Ewald et al., 2008). Gemcitabine was shown to have a better cellular uptake, a longer retention time (Plunkett et al., 1995) and to enhance the antiproliferative capacities of cisplatin in combination regimen treatment (van Moorsel et al., 2000).

While the stereochemical form of natural nucleosides is the β-D-configuration, Troxacitabine is a different kind of pyrimidine analog forming the opposite conformation, the β-l. Its uptake is not mediated by ENTs or CNTs and it is phosphorylated by a different type of kinase, the 3-phosphoglycerate kinase. Troxacitabine’s antiproliferative activity was demonstrated in clinical trials for both solid and hematological malignancies (Swords and Giles, 2007). CNDAC is a cytosine analog with a completely different way of action. In contrast to ara-c, gemcitabine and troxacitabine-mediated cytotoxicity that is achieved through replication fork stalling with concomitant S-phase arrest, CNDAC antiproliferative effects are derived from the capacity to induce G2 arrest and to induce DNA DSBs (Wang et al., 2008). Fludarabine and Cladribine, which are used for the treatment of blood malignancies, represent examples of purine analogs based on modifications of 2′-deoxyadenosine. Fludarabine has a fluorine atom at the 2′ position of adenosine plus a phosphate group at the 5′ carbon of the arabinose ring while the only modification of cladribine is, instead of the fluorine, a chlorine atom in the 2′ site of the sugar. Like their pyrimidine analogs, also these molecules are internalized by the NTs, they undergo the same activation steps and they ultimately kill cells by activating the DDR upon DNA incorporation (Huang et al., 1990). Fludarabine and Cladribine were reported to also interfere with the activity of ribonucleotide reductase, DNA ligase, DNA primase (Clarke et al., 2001) and to induce apoptosis through APAF-1 (Genini et al., 2000). Of note, both drugs result cytotoxic also for non-dividing cells (Galmarini et al., 2002).

Clofarabine is another purine analog that was developed in order to ameliorate the two aforementioned predecessors and it was brought into use in 2006 for the treatment of pediatric acute lymphoblastic leukemia (ALL; Bonate et al., 2006). It carries a fluorine atom at the 2′ site of the purine which increases the stability of the molecule and, like gemcitabine and fludarabine, the triphosphate form of clofarabine blocks DNA synthesis, inhibits ribonucleotide reductase and triggers apoptosis by directly affecting the release of cytochrome c from the mitochondria (Ewald et al., 2008). In addition, clofarabine showed *in vitro* cytotoxicity also in non-small cell lung, colon, central nervous system, ovarian, renal, prostate, and breast cancer cell lines (Bonate et al., 2006).

### Alkylating Agents

Alkylating agents are one of the oldest antineoplastic drugs. The first glimpse of a therapeutic potential of this class of compounds appeared during the first world war when it was noticed that people exposed to sulfur mustard, a chemical warfare, were developing bone marrow suppression and lymphoid aplasia (Krumbhaar and Krumbhaar, 1919). In 1949, Chlormethine, sold under the name of Mustargen, was the first alkylating agent to be approved by FDA for the treatment of leukemia and lymphomas. Alkylating drugs function during all phases of the cell cycle via formation of reactive intermediates, which attack nucleophilic groups on DNA bases with high negative potential. Of consequence, the primary targets of alkylating agents are purines with N7- and O6-methyl guanine being the most stable *in vitro* methylation adducts (Kondo et al., 2010). Base alkylation can also occurs on adenines on positions N1, N3, N6, N7. Pyrimidines can as well be alkylated: cytosines on positions N3 and O2 and thymidines on O2, N3, and O4 sites (Puyo et al., 2014). Alkylation of oxygen atoms can be highly mutagenic, while *N*-akylations are more cytotoxic. RNA, proteins, and lipids can also be targets of alkylation. Alkylating agents can be either mono- or bifunctional depending on the number of active sites they have and the possibility to react with one or two DNA strands. Monoalkylating agents transfer one alkyl group to their targets resulting in a single base modification and, if not promptly repaired, lead to relative base mispairing (alkylated guanines can wrongly pair with thymines) or to strand breakage due to the formation of an apurinic/apyrimidinic (AP) site. On the other side, the two electrophilic sites of bifunctional agents can attack two different bases on the same or on opposite DNA filaments to form intra- or interstrand crosslinks, respectively, which potentially inhibit strand separation during replication or transcription.

DNA crosslinks can also be introduced as a result of the interaction between two adjacent bases previously modified by monofunctional agents (Fu et al., 2012). Alkylating agents used in chemotherapy are divided in six groups: nitrogen mustards, alkyl sulfonates, ethylenimines, triazines, and nitrosoureas.

Nitrogen mustards represent the oldest group of bifunctional alkylating agents initially used to treat cancer patients. Due to the short half-life and high toxicity, the use of chloremethine, the progenitor of this class of compounds, is actually restricted to veterinary medicine but many of its derivatives were developed and are actually applied in the treatment of different neoplasias. Chlorambucil and Bendamustine are used for treating chronic lymphocytic leukemia (CLL). Melphalan, apart from being implied in breast and ovarian cancers, Hodgkin’s disease and neuroblastoma, is the standard treatment, in combination with prednisone, for multiple myeloma (Alexanian et al., 1967).

Cyclophosphamide, the most used drug of this class of agents, possesses the broadest spectrum of anticancer activity. In addition to its beneficial role in hematological malignancies, it is also effective in the treatment of solid tumors like bladder, brain, breast, cervix, endometrium, lung, ovary, and testis (Emadi et al., 2009). Ifosfamide is structurally similar to cyclophosphamide and it is as well utilized in solid tumors such as cervix, testes, head and neck, breast, ovary, and lung tumors. Cyclophosphamide and ifosfamide are prodrugs that require activation in the liver by cytochromes p450.

Busulfan belongs to the class of alkyl sulfonates and is one of the most important bifunctional agent for the cure of chronic myelogenous leukemia (CML; Haut et al., 1961), lymphomas and myeloproliferative disorders.

Thiotepa and altretamine are examples of another class of bifunctional alkylating agents, ethyleneimines. The first one is used for ovarian, breast, and bladder cancer (van Maanen et al., 2000), while the second one has shown positive effects for recurrent ovarian cancer following cisplatin therapy (Chan et al., 2003).

Triazines and nitrosoureas represent two classes of monofunctional alkylating agents with the main difference in their donor alkyl group: a methyl for triazines and chloroethyl for nitrosoureas. Examples of triazines are dacarbazine, an hepatic activable agent included in the treatment of melanoma (Hersh et al., 2011) and temozolomide which is used for primary brain tumors thanks to its high bioavailability in the nervous system (Stupp et al., 2005). Nitrosoureas reduce the *in vitro* proliferation of different cancer cell lines (Gnewuch and Sosnovsky, 1997) and possess activity against solid and non-solid tumors. Carmustine, lomustine, nimustine, and fotemustine are examples of nitrosoureas derivatives that need to be considered for the treatment of brain tumors and skin cancer.

The classic negative side effects of alkylating agents are nausea and fatigue as well as myelo- and immunosuppression and cardiac dysfunction. In addition, most of these chemotherapy agents have mutagenic and carcinogenic potential.

The products of mono *N*-alkylation are repaired by base excision repair (BER) or direct reversal. BER is initiated by DNA glycosylases, which recognize and remove the DNA lesion with the concomitant formation of an abasic (AP) site. The AP site is then processed by specific endonucleases and the missing nucleotide is inserted by DNA polymerase-β. Sealing of the nick is performed by DNA ligase, which finally restores the DNA integrity (Kim and Wilson, 2011). The BER pathway specifically repairs N7MeG, N3MeA, and N3MeG and downregulation of BER components [APE1 endonuclease, polymerase-β, poly (ADP-ribose) polymerase (PARP)] was shown to sensitize tumors to alkylating agents (Liu and Gerson, 2004).

The human AlkB homologs ABH2 and ABH3 are demethylases that catalyze the direct reversal of the following lesions: N1MeA, N3MeC, N3MeT, and N1MeG (Aas et al., 2003). Like for BER deficiency, inhibition of AlkB proteins enhances the chemotherapeutic effects of alkylating drugs (Ralhan and Kaur, 2007). Alkylations of the oxygen atoms, on the other hand, are targets of the repair protein methylguanine DNA methyltransferase (MGMT), which is able to transfer the inserted alkyl groups into its own active site in an auto-inactivating reaction (Pegg et al., 1994). MGMT importance is underlined by the notion that *mgmt* deficient cells are more sensitive than wild type to methylating agents (Day et al., 1980) while MGMT overexpression correlates with resistance to temozolomide (Kaina et al., 2007). MGMT is an optimal candidate to be taken in consideration to sensitize alkylating agent-resistant cancers. In this regard, inhibitors like O6-benzyl guanine (O6-BG), a pseudosubstrate of MGMT, have been proved to enhance the response to temozolomide in cells with high level of MGMT (Zhang et al., 2009b).

*O*-alkylations can also be repaired by NER or MMR. In contrast to MGMT, MMR presence is indispensable for the antiproliferative activity of alkylating agents: in MMR deficient cells, the damage accumulates but is not translated in the apoptotic signal. Abrogation of MMR rescues the sensitivity of *mgmt* ^–/–^ mice to *N*-methyl-*N*-Nitrosourea (Klapacz et al., 2009).

All the aforementioned repair systems act together with HR, FA, and TLS pathways to solve the more complex lesions caused by the action of bifunctional alkylating agents. The interstrand DNA crosslinks introduced by the latter are usually repaired previous transformation in DSBs (Kondo et al., 2010). Targeting key proteins involved in these processes could represent an attractive strategy to enhance the tumor response to this class of chemotherapeutic drugs.

### PARP1 Inhibitors

Personalized medicine uses targeted therapies on specific patients cohorts and PARP1 inhibitors represent a new promising class of chemotherapeutic drugs adopted to exclusively disrupt PARP1 function in HR-defective cancers.

PARP1 belongs to a family of 17 ADP-ribosyltransferases which utilize nicotinamide adenine dinucleotide (NAD^+^) molecules as a substrate to form polymers of ADP ribose units (PAR) on target proteins. This post-translational modification, known as PARylation (Chambon et al., 1964), is a reversible fundamental process of the DDR necessary for recruiting to the damaged site PAR-binding factors involved in chromatin architecture and DNA repair. PARP1 is the most expressed member of the family, it has nuclear localization and it plays a major role in BER by associating with SSBs and recruiting crucial repair proteins like X-ray repair cross-complementing protein 1 (XRCC1; Rouleau et al., 2010). In addition, PARP1 is part of the HR and NHEJ machineries thanks to the interactions respectively with MRE11, RPA, RAD51 (Bryant et al., 2009), and ligase IV (Li et al., 2013).

Synthetic lethality is the phenomenon by which combinations of mutations in two or more genes is lethal whereas single mutation of only one is compatible with viability (Reinhardt et al., 2009).

PARP1 inhibition was found to be effective in the treatment of tumors carrying mutations in BRCA1 or BRCA2 genes. In these tumors, the accumulation of SSBs, upon treatment with PARP1 inhibitors, leads to stalling of replication forks and to the formation of DSBs, which cannot be repaired in the absence of functional BRCA1 and/or BRCA2 proteins finally resulting in high level of genomic instability and eventually cell death. Thus, by exploiting the concept of synthetic lethality, PARP1 inhibitors selectively kill malignant cells that are HR deficient (Rouleau et al., 2010). Since PARP1 dissociation from DNA is mediated by auto-PARylation, PARP1 inhibitors exert their cytotoxic effects also by causing a permanent bound of PARP1 to SSBs thereby inhibiting the accessibility of other PARP proteins to the DNA lesion (Elvers et al., 2011).

Besides BRCA1 and BRCA2, sensitivity to PARP1 inhibitors was also observed *in vitro* for the deficiency of other HR genes including RAD51, RAD54, DSS1, RPA1, NBS1, ATR, ATM, CHK1, CHK2, FANCD2, FANCA, and FANCC (McCabe et al., 2006). This finding support the notion that BRCA associated cancers respond to PARP1 inhibitors due to abnormal HR and indicate this therapy as a possible treatment for all of the tumors displaying features of “BRCAness.” Olaparib was the first PARP1 inhibitor to be approved by the US FDA for the treatment of ovarian cancers with BRCA mutations but many others (e.g., Iniparib, Rucaparib, Niraparib, Veliparib, and BMN-673) are currently assessed in clinical trials, alone or in combination with either chemo or radiotherapy, for several “non-BRCA” tumors (Tangutoori et al., 2015).

### Radiotherapy

Together with surgery and chemotherapy, radiotherapy represents a common treatment option for 50% of cancer patients (Delaney et al., 2005). By releasing large amounts of energy that can be adsorbed by atoms or molecules, IR can directly damage the chemical structure of genetic material and it is consequently used to block cancer cells proliferation and inducing cell death (Jackson and Bartek, 2009). Radiotherapy is given alone or in combination with chemotherapy (chemoradiotherapy) or before (neoadjuvant treatment), during (concurrent treatment) and after surgery (adjuvant treatment) and it can be delivered on patients either with external devices or, internally, with sealed radioactive sources placed inside the body near the tumor area (brachytherapy; Baskar et al., 2012). Unsealed radiation sources (such as iodine, phosphorus, strontium, or samarium), sometimes bound to an antibody directed to the malignant cells, represent the last method to deliver IR in tumor therapy. This class of radiopharmaceuticals drugs are present in liquid forms and usually administered orally or by vein injection (Wallner, 2006).

Apart from being used for curing, radiotherapy can also be adopted with palliative intent to release the pain associated with specific types of cancer.

Photons (X-rays and gamma rays) and charged particles are the main forms of IR utilized in cancer therapy. X- and gamma rays represent widely used photon beams with low radiation charge generated respectively from electrons exciting devices and from the decay of radioactive substances like caesium, cobalt or radium. Once they enter the body, electromagnetic waves of photons do not stop on their targets but they keep going and affecting the surrounding healthy tissues by interacting with the electrons of other molecules. Moreover, the radiation dose decreases as the depth of penetration in the body increase (Hall and Giaccia, 2011).

In photon therapy, most of the DNA damage is inflicted indirectly by the reaction with free radicals species formed upon ionization of water components. Of consequence, the availability of oxygen becomes one of the major limitations in treating solid tumors that are known to be hypoxic. To overcome this problem, chemical radiosensitizer that can react with free radicals in a similar way to oxygen have been developed. Nimorazole and Sanazole represent the best examples of oxygen mimicking drugs actually adopted in the clinic (Lomax et al., 2013).

Charged particles radiation therapy use cyclotron and synchrotron to accelerate electrons, protons or heavy ions like carbon causing direct DNA damage due to the higher linear energy transfer (LET) capacity. The large mass of protons and other charged particles, and their unique absorption profile (Bragg’s peak: maximum release of energy when the particles stop traveling through the body) minimize the lateral side scatter and inflict a more precise damage to the target (Allen et al., 2011). Although radiotherapy is one of the most effective ways to kill a cancer cell, it causes both early (acute) and late (chronic) side effects due to killing of normal cells and triggering inflammatory responses. Fatigue and sore skin are the most common acute side effects while the chronic ones largely depends on which part of the body is treated with the possibility to develop secondary cancers. Technological advances, like the use of image-guided (IGRT) or intensity-modulated radiotherapy (IMRT), have made a great progress in precisely delivering IR to patients without affecting healthy tissues but the effectiveness of the treatment does not rely only on this aspect. Other factors, such as the genetic background of the patient, have to be considered to maximize the benefit of radiotherapy (Thoms and Bristow, 2010). As mentioned before, IR attacks directly or indirectly the DNA molecule inflicting lesions that range from abasic site to the more cytotoxic SSBs and DSBs (Wallace, 2002). DNA damage sensing and repair mechanisms, and their status within a specific tumor subtype, are therefore of great importance in the establishment of cancer cell sensitivity to radiotherapy and for assessing how their modulation can be exploited in chemoradiotherapy. Inhibition of cell cycle checkpoints mediated by CHK1 and CHK2 or proteins involved in BER, such as APE or POLQ, or in DSBs repair, like ATM or DNA-PK, have indeed been shown to sensitize cancer cells to radiotherapy (Begg et al., 2011).

## Concluding Remarks

DNA damage occurs on a daily basis by endogenous and exogenous sources. Distinct DNA repair systems recognize and remove the lesions. When the damage remains unrepaired DNA damage checkpoints can halt the cell cycle or induce cellular senescence or apoptosis. Erroneous repair or replicative bypass of lesions can result in mutations and chromosomal aberrations. When mutations affect tumor suppressor genes or oncogenes, cell might transform into cancer cells. Therefore, DNA repair is essential for preventing tumor development. However, once a cancer has developed, DNA damage can be exploited to reduce cancerous growth and evoke apoptotic demise of cancer cells. Thus, chemo- and radiotherapies are still today, over 60 years after having been first introduced into tumor therapy, important strategies to fight cancer. Given the central role of genome instability in triggering and treating cancer, it is likely that genotoxic treatments will remain an important avenue of cancer therapy. Also the better understanding of DNA repair systems will allow therapies that specifically target selected repair pathways. It will be of particular importance to gain a deeper understanding how the various DNA repair systems interact with each other in the context of cellular homeostasis and DNA metabolism in order to optimize targeted approaches to cancer therapy.

### Conflict of Interest Statement

The authors declare that the research was conducted in the absence of any commercial or financial relationships that could be construed as a potential conflict of interest.
